# Effect of modified atmosphere packaging on preservation property and microbial communities of golden pompano (*Trachinotus ovatus*) fillets during 0 °C storage

**DOI:** 10.1016/j.fochx.2026.104091

**Published:** 2026-06-11

**Authors:** Yisheng Huang, Zewei Zhang, Bin Zhang, Chang Liu, Yuzhong Zheng, Hui Zhu, Laihao Li, Wanling Lin

**Affiliations:** a School of Life Sciences and Food Engineering, Hanshan Normal University, Chaozhou 521041, China; b Guangdong Key Laboratory of Functional Substances and Health Products from Medicinal Edible Resources, Chaozhou 521041, China; c Key Lab of Aquatic Product Processing, Ministry of Agriculture and Rural Affairs, South China Sea Fisheries Research Institute, Chinese Academy of Fishery Sciences, Guangzhou 510300, China

**Keywords:** *Trachinotus ovatus*, Modified atmosphere packaging, Preservation property, Microbial community

## Abstract

The effects of air packaging (AP) and modified atmosphere packaging 1 (MAP1) (50% CO₂/50% N₂), MAP2 (70% CO₂/30% N₂), and MAP3 (90% CO₂/10% N₂) on the preservation property of Golden Pompano *Trachinotus ovatus* stored at 0 °C were studied. Results demonstrated that MAP exhibited better preservation effects than AP. Based on sensory scores, the shelf life of fillets under AP and MAP was 10 and 16 days, and MAP2 was the best among all treatments (*P* < 0.05). MAP significantly inhibited increases in total volatile basic nitrogen (TVB-N), thiobarbituric acid (TBARS), K-value, and total viable count (TVC), slowed reductions in hardness and springiness (*P* < 0.05). Proteobacteria was the dominant phylum, *Exiguobacterium*, *Vibrio* and *Citrobacter* were the prevalent population in MAP at the end of storage. *Vibrio anguillarum* was the predominant taxon associated with spoilage. In summary, MAP2 demonstrates considerable potential for enhancing the shelf life of *T. ovatus* at 0 °C.

## Highlights

• MAP significantly extends the shelf life of Golden Pompano fillets at 0°C.
• *Vibrio anguillarum* was the predominant taxon associated with spoilage.
• Integrated analysis links microbial dynamics to physicochemical and sensory changes.
• 70% CO₂/30% N₂ optimally inhibits quality deterioration by suppressing microbial growth and biochemical degradation.

## 1 Introduction

Golden pompano (*Trachinotus ovatus*), a high-value marine fish within the family Carangidae, is prized for its tender white flesh, pleasing flavor, and nutritional profile characterized by abundant proteins and omega-3 polyunsaturated fatty acids (PUFAs)(Li et al., 2016). As an important fish species across Asia-Pacific markets, it provides essential amino acids (Kou et al., 2015) and polyunsaturated fatty acids (PUFAs) (Sun et al., 2016), driving substantial consumer demand for premium seafood products.

Current postharvest handling methods, including fresh, chilled, and frozen formats, are constrained by limited shelf stability, thereby diminishing the economic value of golden pompano products (Qiu et al., 2014). Quality deterioration arises from synergistic microbial proliferation (Li et al., 2020; Pazdzior et al., 2023; Tsoukalas et al., 2023) and biochemical degradation pathways (Huang et al., 2023; Lang et al., 2023; Yu et al., 2021), manifesting as texture softening, discoloration, and moisture loss (Lang et al., 2023). These alterations frequently downgrade fish to low-value byproducts like animal feed or fertilizer, underscoring the urgent need to develop sustainable preservation technologies. To ensure the quality and market value of *T. ovatus* while addressing consumer preferences for fresh and safe seafood, it is imperative to establish sustainable and cost-effective postharvest preservation techniques that effectively prolong shelf life.

Modified atmosphere packaging (MAP) preservation technology has been widely used in aquatic preservation (Babic Milijasevic et al., 2023; Zhang et al., 2022). MAP utilizes high-barrier films to encase food products within a customized gas environment, predominantly composed of carbon dioxide (CO_2_) and nitrogen (N_2_) gas, which effectively suppresses microbial proliferation (Qiu et al., 2019), reduces the enzymatic reaction and retards the rate of lipid oxidation (Bassey et al., 2021). CO₂ serves as the primary antimicrobial component in MAP systems targeting fish spoilage microorganisms. Empirical evidence confirms that high CO_2_ levels extend the shelf life of fish and fish products due to the inhibition of microbial growth (Esteves et al., 2021; Tan et al., 2025; Yang et al., 2023). MAP with high levels of CO_2_ generally promotes fresh fish storage stability (Babic Milijasevic et al., 2023; Olatunde & Benjakul, 2018). Milijasevic et al. found that MAP have great potential for preserving fish quality and extending the shelf life of gutted rainbow trout from 13 days in 40% CO_2_/60% N_2_ and 60% CO_2_/40% N_2_ to 16 days in 90%CO_2_/10%N_2_ (Babic Milijasevic et al., 2023). In addition, the strategic replacement of oxygen (O₂) with inert N₂ in MAP effectively suppresses aerobic spoilage bacteria and mitigates lipid oxidation (Sivertsvik et al., 2002). In the MAP trial, the shelf life of fish are usually assessed through comprehensive analysis of multiple quality indicators, including microbial profiles (TVC, 16S rRNA), chemical properties (TVB-N, K-value), physical characteristics, and sensory attributes, etc. (Esteves et al., 2021; Goes et al., 2025).

While previous researchers have investigated the quality changes of *T. ovatus* fillets under superchilling conditions (−3 °C) (Zhang et al., 2022), a critical research gap remains regarding their quality evolution and microbial succession at 0 °C, the most representative temperature for actual retail display and cold-chain transportation. In contrast to sub-zero superchilling, which mainly inhibits microbial activity by forming ice crystals, 0 °C storage represents a realistic commercial scenario where spoilage microorganisms remain metabolically active and texture maintenance becomes particularly challenging. To address this gap, the present study comprehensively evaluated the effects of air packaging (AP) and three MAP regimes (50% CO₂/ 50% N₂, 70% CO₂ / 30% N₂, and 90% CO₂ / 10% N₂) on the preservation properties and microbial communities of *T. ovatus* fillets stored at 0 °C. By integrating sensory evaluation, physicochemical indices, and total viable counts (TVC) with high-throughput 16S rRNA sequencing, this study elucidates the interplay between gas composition, microbial dynamics, and quality deterioration. The findings aim to provide both practical guidelines for the aquatic industry and mechanistic insights into MAP preservation at commercial refrigeration temperatures.

## 2 Materials and methods

### 2.1 Samples and chemicals

Fresh *T. ovatus* (mean weight: ∼510 g; length: ∼25 cm) were purchased from a local aquatic market in Chaozhou, China. Harvested fish were immediately placed on ice flakes and transported to the laboratory within 1 h. Upon arrival, the fish were beheaded, scaled, gutted, and filleted, and then washed immediately with cold sterile water. The fillets were then drained at 0 °C for 3 min and prepared for preservative packaging.

The fish fillets (72 ± 4 g, approximately 10 × 5 × 1.5 cm^3^) were randomly assigned to four modified atmosphere packaging (MAP) treatments: (a) 100% air, (b) 50% CO₂/50% N₂, (c) 70% CO₂/30% N₂, and (d) 90% CO₂/10% N₂. For each treatment, three independent packages were prepared as biological replicates for each of the nine sampling time points (days 0, 2, 4, 6, 8, 10, 12, 14, and 16). Each package contained six fillets and was sealed using a gas packaging machine (Luodiboer, China). All packages were stored in a constant temperature and humidity chamber at 0 ± 0.5 °C and 90 ± 5% relative humidity. At each designated sampling time, three independent packages per treatment (*n* = 3) were retrieved entirely for destructive analysis.

### 2.2 Gas analysis

The gas composition in the headspace of all MAP samples was monitored using a Headspace Gas Analyzer IN-DK3 O₂/CO₂/N₂ (Laiying Optoelectronics Technology Co., Ltd., Shandong, China). Measurements were acquired by penetrating the package septum with a needle prior to opening, and this procedure was conducted before each sampling event.

### 2.3 Sensory evaluation

The sensory quality of *T. ovatus* was evaluated following the methodology described by Cardenas(Cardenas Bonilla et al., 2007) with minor adaptations. A trained panel of 10 assessors participated in three 1-h training sessions prior to formal evaluation. During training, assessors were familiarized with the scoring scale using reference samples representing different freshness levels (e.g., fresh fillets for score 10, spoiled fillets for score 1). Consensus on attribute definitions (color, odor, texture, and overall acceptability) was established to ensure inter-assessor reliability. Each of the four parameters was rated on a 10-point scale (1 = poorest quality; 10 = optimal quality). The individual scores were summed to obtain a total sensory score, resulting in a theoretical range of 4 to 40. Fish samples were deemed unacceptable when the total sensory score fell below 20. This sensory evaluation study did not require formal ethical approval according to the guidelines. The assessment only involved non-invasive sensory evaluation of chilled golden pompano fillets by trained panelists, with no health or safety risks to participants. All panelists provided verbal informed consent prior to participation, and all sensory data were recorded anonymously to protect privacy.

### 2.4 TBARS

The determination of TBARS was carried out according to the Chinese National Standard GB5009.181–2016, 5 g of fish sample was accurately weighed into a 100 mL stoppered conical flask, followed by the addition of 50 mL of 7.5% (*w*/*v*) trichloroacetic acid (TCA) mixture. The mixture was shaken well, capped, and sealed, then oscillated on a constant-temperature shaker at 50 °C for 30 min. After cooling to room temperature, the mixture was filtered through double-layer quantitative slow filter paper, and the filtrate was collected for use. For TBARS testing, 5 mL filtrate and 5 mL of each TBARS standard series solution were separately transferred into 25 mL stoppered colorimetric tubes. A sample blank was prepared by adding 5 mL of TCA mixture instead of the sample filtrate. Subsequently, 5 mL of TBA aqueous solution was added to each tube, which was then capped, mixed, and reacted in a 90 °C water bath for 30 min. After cooling to room temperature, the absorbance of each solution was measured at 532 nm using a 1 cm path length cuvette, with the sample blank used for zero adjustment. A standard curve was constructed with TBARS concentrations as the x-axis and absorbance values as the y-axis, based on which the TBARS content of the sample was calculated.

### 2.5 TVB-n

TVB-N content was determined following the Chinese National Standard GB 5009.228–2016. Specifically, 10 g of fish sample was homogenized with 75 mL of distilled water in a conical flask for 30 min, then filtered. A 5 mL aliquot of the filtrate was mixed with 5 mL of MgO suspension in a reaction chamber. Subsequently, 10 mL of boric acid solution and a mixed indicator (methyl red: methylene blue, 2:1 *v*/v) were added, followed by steam distillation. The resulting distillate was titrated with 0.1 mol/L HCl solution. TVB-N values were calculated from the HCl titration volume and expressed as mg of nitrogen per 100 g (mg/100 g) of sample.

### 2.6 Total viable counts

A 25-g sample of *T. ovatus* fillets was aseptically transferred into a sterile stomacher bag and homogenized with 9 volumes of sterile physiological saline (0.85% NaCl) using a stomacher for 1 min. Serial decimal dilutions were subsequently prepared from the primary homogenate. Total viable counts (TVC) were determined by plating appropriate dilutions on agar, incubating at 30 °C for 48 h, and expressed as log10 colony forming units (CFU)/g.

### 2.7 K-value

K-value was determined according to methods proposed by Karim et al. (Karim et al., 2019). ATP-related compounds were analyzed using HPLC (1260 infinity, Agilent, USA). K-value was calculated as follows:

(1) $$K\;\text{value}\left(\%\right)=\frac{\mathrm{HxR}+\mathrm{Hx}}{\mathrm{ATP}+\mathrm{ADP}+\mathrm{AMP}+\mathrm{IMP}+\mathrm{HxR}+\mathrm{Hx}}\times 100$$

### 2.8 Texture

Samples of uniform size were excised from the same dorsal region of *T. ovatus and* analyzed using a texture analyzer (TMS-Pro; Beijing Yingsheng Hengtai Technology Co., Ltd.). The instrument parameters were set as follows: a P6 probe (6 mm diameter), test speed of 1 mm/s, compression ratio of 30%, test interval of 5 s, and automatic trigger force of 0.04 N. Measurements were performed with 3 replicates per group, and the mean value was calculated. Each group was tested in triplicate.

### 2.9 Histological microstructures

Samples for microstructure analysis were prepared for microscopic examination following standard hematoxylin-eosin (HE) staining protocols (Cardenas Bonilla et al., 2007), with modifications adapted for *T. ovatus* to visualize extracellular spaces and muscle fiber shrinkage. Following deparaffinization and rehydration, 5-μm cross-sectional tissue sections were processed through a series of staining steps. First, sections were stained with hematoxylin solution for 5 min, briefly differentiated by dipping 5 times in 1% acid ethanol (1% HCl in 70% ethanol), and then rinsed with distilled water. Next, sections were stained with eosin solution for 3 min, followed by dehydration through a graded ethanol series (70%–100%) and clearing in xylene. Finally, the prepared slides were examined and imaged using an Olympus BX53 optical microscope (Tokyo, Japan) equipped with a digital camera system.

### 2.10 Isolation and identification of spoilage bacteria

Under aseptic conditions, approximately 25 g of tissue sample was aseptically excised from the fish using sterilized tools. The sample was transferred into a sterile homogenization bag containing 225 mL saline, followed by homogenization in a stomacher at 10 strokes/s for 60 s to achieve a homogeneous milky suspension. Serial dilutions (10^−4^, 10^−5^, 10^−6^) were prepared, and 100 μL aliquots were spread onto five selective media: PCA, IA, TSA, PBA (incubated at 30 °C for 48 h), and MRS (37 °C for 48 h). Typical colonies were selected and streaked onto fresh media for purification via repeated streaking (≥3 rounds). Preliminary bacterial identification was conducted through systematic evaluation of colony morphological characteristics and biochemical parameters, following Bergey's Manual of Systematic Bacteriology and Manual of Methods for General Bacteriology.

Genomic DNA was extracted using the MO BIO Laboratories kit (Beijing, China) for subsequent 16S rDNA analysis following the manufacturer's instructions and stored at −80 °C. For 16S rRNA gene amplification, forward primer 27f (5′-GAGAGTTTGATCCTGGCTCAG-3′) and reverse primer 1492r (5′-CTACGGCTACCTTGTTACGA-3′) were used. The PCR reaction system (50 μL total volume) included: 2× Rapid Taq Master Mix (containing buffer, dNTPs, Taq enzyme) (25 μL), template DNA (2 μL), forward and reverse primers (each 2 μL), and sterile ddH₂O (21 μL). The cycling conditions were as follows: initial denaturation at 94 °C for 5 min, followed by 33 cycles of 94 °C for 1 min (denaturation), 52 °C for 1.5 min (annealing), and 72 °C for 1.5 min (extension), with a final extension at 72 °C for 10 min.

PCR products were visualized via 1% agarose gel electrophoresis. Successful amplification fragments were purified and sent to Tianyi Huiyuan Gene Technology Co., Ltd. (Shanghai, China) for Sanger sequencing. The obtained sequences were BLAST-analyzed against the NCBI database, and sequences with >99.5% similarity were used for species identification. Final bacterial species were determined by integrating morphological and molecular identification results, following *Bergey's Manual of Systematic Bacteriology* and *Manual of Methods for General Bacteriology*.

### 2.11 DNA extraction and high-throughput sequencing

Total genomic DNA was purified from fillet samples using a commercial DNA extraction kit (MO BIO Laboratories, Beijing, China) following the supplier's protocol. DNA aliquots were cryopreserved at −80 °C prior to amplification of the 16S rRNA V3-V4 hypervariable region using universal primers 338F (5′-ACTCCTACGGGAGGCAGCA-3′) and 806R (5′-GGACTACHVGGGTWTCTAAT-3′). PCR reactions (20 μL volume) comprised: 13.25 μL H₂O, 2.0 μL 10× ExTaq Buffer, 0.5 μL template DNA (100 ng/μL), 1.0 μL each primer (10 μM), 2.0 μL dNTP mix, 0.25 μL ExTaq polymerase (5 U/μL). Thermocycling conditions: 95 °C/5 min (initial denaturation); 25 cycles of 95 °C/30 s, 50 °C/30 s, 72 °C/40 s; final extension at 72 °C/7 min. Amplicons were electrophoresed on 1.8% agarose gels, purified with VAHTSTM DNA Clean Beads (Vazyme, China), and quantified via Quant-iT™ dsDNA Assay (Thermo Fisher, USA). Illumina Sequencing & Bioinformatic Processing Libraries were sequenced on an Illumina HiSeq 2500 platform (250 bp paired-end reads; Biomarker Technologies, China). Each group comprised three independent samples.

Raw Reads Processing Workflow: a) Read Merging: Paired-end reads were merged into raw tags using FLASH (v1.2.7, http://ccb.jhu.edu/software/FLASH/). b) Quality Control: Low-quality sequences were filtered using QIIME v1.7.0 (via split_libraries_fastq.py, http://qiime.org/scripts/split_libraries_fastq.html). Chimeric sequences were identified and removed using the UCHIME algorithm, http://www.drive5.com/usearch/manual/uchime_algo.html). c) OTU Clustering: High-quality sequences were clustered into operational taxonomic units (OTUs) at a 97% similarity threshold using UCLUST (v1.2.22, http://www.drive5.com/usearch). d) Taxonomic Annotation: The RDP Classifier (http://sourceforge.net/projects/rdp-classifier/)) was employed to assign taxonomy against the SILVA database (Release 119, http://www.arb-silva.de) with a confidence threshold of 0.8. Community Analysis: a) Alpha Diversity: Metrics including Chao1, ACE, Shannon index, and Good's coverage were calculated using Mothur (v1.30, http://www.mothur.org/). b) Beta Diversity: Principal Coordinates Analysis (PCoA) and hierarchical clustering were performed based on weighted UniFrac distances (analyzed in R v2.15.3). c) Heatmaps: Genus-level relative abundances were visualized using R software.

### 2.12 Statistical analysis

Data were analyzed using one-way ANOVA and are presented as means ± standard deviation (SD) from triplicate independent biological replicates. Normality was assessed using the Shapiro-Wilk and Levene's tests. One-way ANOVA with Duncan's multiple range test was used to determine statistical significance, with *P* < 0.05 considered statistically significant (IBM SPSS Statistics 26, IBM Corp., Armonk, NY, USA). For microbial community analysis, inferential statistics were performed to complement the descriptive data. PERMANOVA (Adonis) based on Bray-Curtis dissimilarity was used to determine significant differences in β-diversity. LEfSe analysis (Linear discriminant analysis Effect Size) was conducted to identify biomarker taxa, with an LDA score threshold of >2.0 and a significance level of *P* < 0.05. Correlation analysis and principal component analysis (PCA) were performed using Origin 9 (OriginLab Corp., Northampton, MA, USA). Graphs were generated using GraphPad Prism 9 (GraphPad Software, Boston, MA, USA).

## 3 Results and discussion

### 3.1 Sensory analysis

**Fig. 1:**
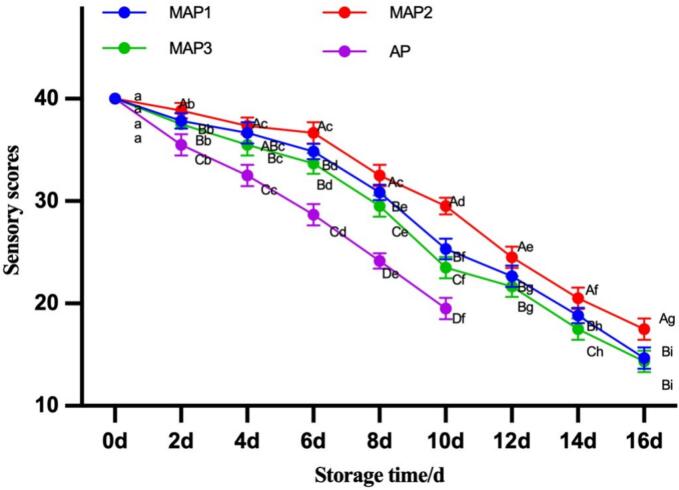
Changes in sensory scores of *Trachinotus ovatus* during cold storage under different modified atmosphere gas. Bars represent the standard deviation (*n* = 3). Different capital letters indicate significant differences within groups and different lowercase letters indicate significant differences between groups (*P* < 0.05).

The progressive decline in sensory scores was primarily driven by microbial growth, endogenous protease activity, and lipid oxidation in muscle tissues. As illustrated in Fig. 1, all experimental groups exhibited a continuous decrease in sensory scores over extended storage periods (*P* < 0.05), consistent with previous reports on Gray Triggerfish (*Balistes capriscus*) (Esteves et al., 2021) and rainbow trout (*Oncorhynchus mykiss*) (Nikzade et al., 2019). Significant differences emerged among treatments, with AP showing substantially lower sensory scores than MAP from day 2 onward (*P* < 0.05). The sensory score of the AP declined to 20.0 by day 10, indicating complete loss of fillet quality and the end of shelf life. This enhanced stability is likely attributable to CO2 and N2 could effectively limited microbial growth and the release of a lipolytic enzyme (Kimbuathong et al., 2020; Zhang et al., 2022). Notably, fillets packaged under 70%CO₂/30%N₂ retained significantly higher sensory scores than those stored with 50% CO₂/50% N₂ or 90% CO₂/10% N₂ from day 6 to 10 (*P* < 0.05), suggesting this gas ratio achieved optimal microbial inhibition and structural integrity preservation during refrigerated storage. This optimal effect of MAP2 (70% CO₂/30% N₂) could be explained by the balance between antibacterial activity and quality protection. A higher CO₂ concentration (90% CO₂) may cause slight acidification in fish muscle and affect texture properties (Qian, Gao, et al., 2025; Qian, Shi, et al., 2025), whereas a lower CO₂ level (50% CO₂) provides insufficient antibacterial inhibition(Sai-Ut et al., 2025). The gas ratio of 70% CO₂/30% N₂ not only ensures strong antimicrobial effects but also avoids quality damage induced by over-high CO₂, thus showing the best preservation performance among all treatments.

### 3.2 TBARS

**Fig. 2:**
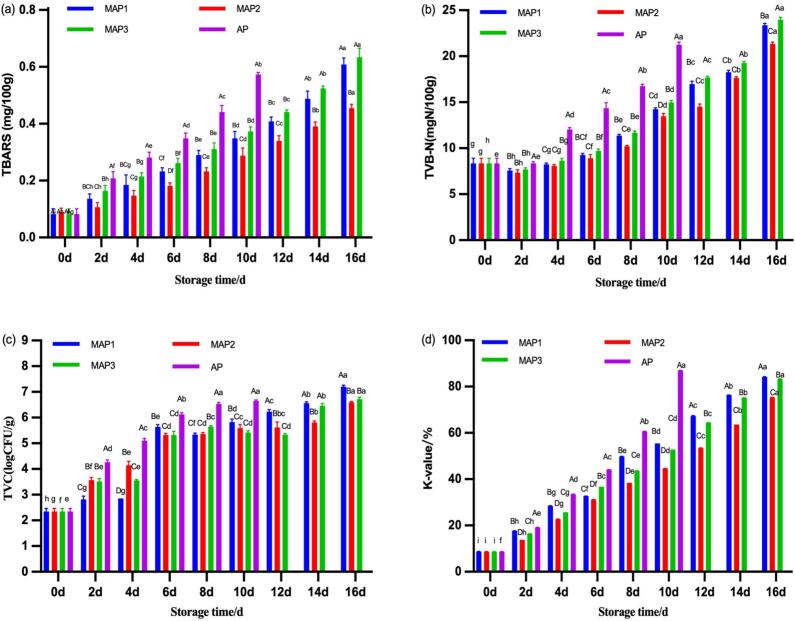
Changes in TBARS (a), TVB-N (b), TVC(c), and K-value(d) of *Trachinotus ovatus* during cold storage under different modified atmosphere gas. Bars represent the standard deviation (n = 3). Different capital letters indicate significant differences within groups and different lowercase letters indicate significant differences between groups (*P* < 0.05).

TBARS index is usually related to the degree of lipid oxidation in tissues, which is an important indicator reflecting the freshness of fish (Kaewprachu et al., 2017). As demonstrated in Fig. 2a, all treatment groups exhibited significant time-dependent increases in TBARS values throughout storage (*P* < 0.05). The progressive lipid oxidation pattern aligns with previous observations in mackerel (*Scomber scombrus*) and Asian sea bass (*Lates calcarifer*), suggesting a universal preservation challenge in marine species. Notably, MAP groups demonstrated superior lipid stability compared to AP storage. AP exhibited the most significant increase from 0.08 to 0.57 mg MDA/kg by day 10, whereas MAP maintained significantly lower final levels: 0.35, 0.29, and 0.38 mg MDA/kg for MAP1, MAP2, and MAP3, respectively. The efficacy of MAP2 in suppressing lipid oxidation is noteworthy, likely attributable to a synergistic dual-action mechanism. This involves the suppression of microbial proliferation via carbon dioxide and the direct inhibition of lipolytic enzyme activity. Consequently, this attenuated TBARs progression in MAP2 suggests that optimized gas combinations can effectively decelerate oxidative degradation pathways in stored fillets.

### 3.3 TVB-n

TVB-N serves as a critical freshness indicator in fish, reflecting enzymatic and microbial protein degradation (Volpe et al., 2015). As illustrated in Fig. 2b, TVB-N values increased progressively across all groups during storage. Notably, the gap in TVB-N values between MAP and AP widened progressively over time, with AP exhibiting significantly higher levels compared to MAP from day 2 onward (*P* < 0.05). This increase corresponds to the accumulation of nitrogenous compounds like ammonia, dimethylamine, and trimethylamine (TMA), with TMA specifically derived from microbial reduction of trimethylamine oxide (TMAO) (Li et al., 2021; Zhuang et al., 2020). Notably, sensory evaluations revealed a pronounced putrid odor by day 10, coinciding with TVB-N nearing organoleptic acceptability thresholds (Jia et al., 2019). Among MAP treatments, the 70% CO₂/30% N₂ mixture demonstrated superior inhibition of proteolytic spoilage, yielding significantly lower TVB-N values than other gas combinations. This suppression likely stems from the synergistic antibacterial activity of CO₂ and N₂, which restricts microbial growth and subsequently mitigates the production of TMA. The observed TVB-N trends thus underscore MAP's efficacy in decelerating protein-derived quality deterioration during storage.

### 3.4 TVC

As illustrated in Fig. 2c, the total viable count (TVC) of all treatment groups exhibited a progressive increase during refrigerated storage. The initial TVC (2.35 log CFU/g) aligned with the high-quality status of *T. ovatus* fillets, given that the microbiological threshold for fresh fish is defined as 5 log CFU/g. Notably, all samples demonstrated statistically significant TVC escalation with storage time (*P* < 0.05), whereas MAP treatments effectively suppressed microbial proliferation. By day 10, the TVC values of MAP1, MAP2, and MAP3 groups were significantly reduced compared to AP, likely attributable to the bacteriostatic effects of CO₂ and N₂. These gases may induce metabolic dormancy and inhibit enzymatic activity in bacterial communities during storage. According to the International Commission on Microbiological Practices in Food (ICMSF), the safety limit for fresh fish is 7 log CFU/g (Liu et al., 2023). In the present study, AP reached 6.65 log CFU/g by day 10, while MAP exhibited delayed microbial growth, attaining 7.20, 6.59, and 6.71 log CFU/g at day 16. This divergence suggests that the synergistic antioxidant and antimicrobial properties of CO₂/N₂ effectively decelerated spoilage kinetics. Consequently, MAP technology extended the shelf life of *T. ovatus* fillets, with TVC serving as a robust microbiological criterion for quality assessment.

### 3.5 K-value

The K-value is deemed a critical indicator for fish freshness evaluation, which is derived from the ATP degradation pathway ATP → ADP → AMP → IMP→HxR → Hx, reflects flavor evolution in fish muscle (Febrianto & Zhu, 2023). While IMP contributes umami taste, its conversion to bitter-tasting hypoxanthine derivatives (HxR/Hx) correlates with quality deterioration (Yu et al., 2021). As illustrated in Fig. 2d, all *T. ovatus* samples exhibited progressive K-value increases during storage (*P* < 0.05), consistent with TBA and TVB-N trends. Defined as the (HxR + Hx)/Σ(ATP degradation products) ratio (Chang et al., 2020), K-values exceeding 60% signal spoilage onset, with 20–40% indicating acceptable freshness (Cai et al., 2014). MAP treatments significantly attenuated K-value progression compared to AP. Initial K-values averaged 8.64%, confirming high freshness. By day 6, air-stored fillets surpassed the 40% threshold, while MAP groups maintained lower levels: 32.60% (MAP1), 31.17% (70% CO₂/30% N₂), and 36.71% (50% CO₂/50% N₂). The MAP2 mixture demonstrated superior efficacy, limiting K-values to 44.59% at day 10 versus 55.38% (MAP1), 52.79% (MAP2), and 86.96% (AP). By day 16, this treatment achieved 75.30%, significantly lower than 84.15% (MAP1) and 83.3% (MAP3) (*P* < 0.05). These results substantiated the capacity of CO₂/N₂ mixtures to retard ATP degradation via microbial suppression, thereby preserving sensory quality.

### 3.6 Texture

**Fig. 3:**
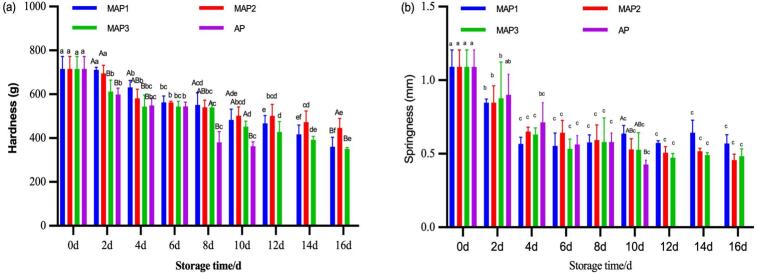
Changes in Hardness (a) and Springness (b) of *Trachinotus ovatus* during cold storage under different modified atmosphere gas. Bars represent the standard deviation (n = 3). Different capital letters indicate significant differences within groups and different lowercase letters indicate significant differences between groups (*P* < 0.05).

Autolysis begins immediately after the death of aquatic products, driven by the redistribution of water within the muscle and the oxidative denaturation of proteins (Thepnuan et al., 2008). The changes in muscle hardness values of golden pompano fillets during storage are shown in Fig. 3. The initial hardness value of fresh fillets was 715.21 ± 57.23 g. The hardness value of fillets decreased sharply with the extension of storage time, with AP reached its lowest value of 363.20 ± 19.64 g at the end of the 10th day. The overall trend of springiness was similar to that of hardness. It decreased sharply during the initial storage period (*P* < 0.05), while no significant changes were observed from day 4 to day 16. The decline in hardness and springiness could be attributed to proteolytic enzyme activity released by microorganisms and structural alterations within the proteins (Thepnuan et al., 2008; Zhang et al., 2022). Wei et al. (2021) pointed out that the reproduction of microorganisms is a main factor in myofibril destruction and quality deterioration (Wei et al., 2021). Protein structure plays a crucial role in determining fish texture, and during storage, protein degradation occurs, resulting in denaturation, structural loosening, and subsequent loss of textural properties (Zhang et al., 2025). The hardness values of MAP and AP differed significantly between days 8 and 10 (*P* < 0.05), and by the end of day 10, the springiness of fillets in the AP group was significantly lower than that in MAP, indicating that MAP containing CO₂ effectively inhibited microbial proliferation in the muscle. Consequently, it not only exerted a beneficial antibacterial effect but also reduced oxidative denaturation of proteins, thereby better preserving the hardness and springiness of fillets during storage. By the end of day 16, MAP-maintained hardness values ranged from 350.03 to 446.04 g, remaining consistently above AP and demonstrating the excellent preservative effect of modified atmosphere packaging on fillet textural quality.

### 3.7 Principal component analysis of quality indicators of golden pompano fillets

#### 3.7.1 Pearson correlation analysis of quality indicators

**Fig. 4:**
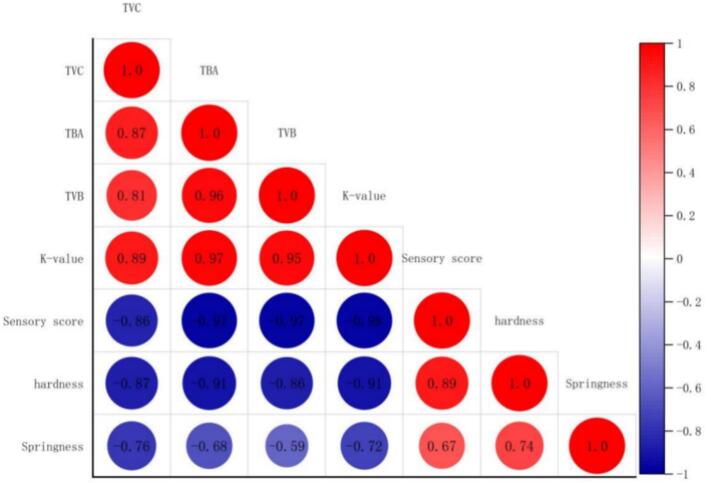
Pearson correlation analysis of quality indicators in golden pompano fillets during storage.

Correlation analysis revealed significant associations between TVC and both physicochemical and sensory parameters (Fig. 4). TVC exhibited strong positive correlations with spoilage biomarkers, specifically TBA (*r* = 0.87), TVB-N (*r* = 0.81), and K-value (*r* = 0.89, *P* < 0.01), indicating that K-value is a highly sensitive predictor of microbial proliferation. Conversely, TVC showed pronounced negative correlations with sensory score (*r* = −0.86) and texture attributes (hardness: *r* = −0.87; springiness: *r* = −0.76). Notably, the absolute correlation coefficients among TBA, TVB-N, K-value, and sensory score were all >0.95 (P < 0.01), demonstrating that protein decomposition, lipid oxidation, and sensory deterioration are intricately linked and collectively dictate the freshness of fillets. The observed strong correlations further support that microbial growth plays a decisive role in quality deterioration. The proliferation of microorganisms promotes the secretion of massive proteases, lipases, and decarboxylases, which directly accelerate protein degradation, lipid oxidation, and ATP catabolism (Wang et al., 2025). These processes consequently lead to the rapid increase in TVB-N, TBARS, and K-value, as well as the decline in textural properties and sensory acceptance. Therefore, microbial growth is the fundamental driving force underlying the quality deterioration of golden pompano fillets during 0 °C storage.

#### 3.7.2 Principal component analysis of quality indicators

The characteristic roots, variance contribution rates and cumulative variance contribution rates of the principal component are presented in Table 1. The principal component analysis (PCA) identified a single component with an eigenvalue of 6.123, which explained 87.467% of the total variance. This result indicates that the quality deterioration of golden pompano fillets under different packaging conditions can be predominantly described by a single composite indicator. By effectively integrating key physicochemical and microbiological parameters, this component allows for a streamlined quality evaluation approach. Practically, this implies that future quality control protocols could rely on a reduced set of proxy indicators highly loaded on PC1 such as K-value or TBA, rather than measuring all indices, to accurately predict the shelf-life of MAP-stored fish fillets.

**Table 1:** Principal component analysis of quality indicator in *Trachinotus ovatus* fillets during storage.

Component^1^	Initial Eigenvalueinitial Eigenvalue			Extract Square and Load		
	Latent Root	Variance Contribution/%	Accumulative Variance Contribution/%	Latent Root	Variance Contribution/%	Accumulative Variance Contribution/%
*1*	*6.123*	*87.467*	*87.467*	*6.123*	*87.467*	*87.467*
*2*	*0.522*	*7.454*	*94.921*			
*3*	*0.162*	*2.309*	*97.230*			
*4*	*0.121*	*1.731*	*98.960*			
*5*	*0.031*	*0.442*	*99.403*			
*6*	*0.025*	*0.352*	*99.754*			
*7*	*0.017*	*0.246*	*100.000*			

1 *Comprehensive indicator formed by data conversion and dimensionality reduction for five quality indicators.*

For the single extracted component, high positive loadings were observed for K-value (0.985), TBA (0.977), TVB-N (0.946), and TVC (0.924), whereas strong negative loadings were found for sensory score (−0.974), hardness (−0.946), and viscosity (−0.779), indicating that these variables were strongly associated with the extracted component (Table 2). In the principal component loading matrix, the absolute values of detection reflect the contribution to the principal component, and the magnitude of contribution in the first principal component was K-value > TBA > sensory score > hardness > TVB-N > TVC > springiness, with K-value having the largest contribution. The principal component in this study mainly reflected the degree of protein hydrolysis, lipid oxidation, and microbial proliferation of fillets. The quality indicators analyzed were defined as TVC (X₁), TBA (X₂), TVB-N (X₃), K-value (X₄), sensory scores (X₅), hardness (X₆), and springiness (X₇). Based on the component score coefficient matrix (Table 3), the linear combination for the first principal component was established as Eq. (2):

(2) Y=0.151×1+0.160×2+0.154×3+0.161×4–0.159×5–0.155×6–0.127×7

**Table 2:** Loading matrix for principal component analysis of quality indicator in *Trachinotus ovatus* fillets during refrigerated storage.

Quality Indicator	Component Coefficient	Coefficient of Component Score
*TVC*	*0.924*	*0.151*
*TBA*	*0.977*	*0.160*
*TVB*	*0.946*	*0.154*
*K-value*	*0.985*	*0.161*
*Sensory score*	*−0.974*	*−0.159*
*Hardness*	*−0.946*	*−0.155*
*Springness*	*−0.779*	*−0.127*

**Table 3:** Identification and colony morphological characteristics of spoilage bacteria in *Trachinotus ovatus* during refrigerated storage.

Storage Times	Bacterial species	Code	Quantity	Characteritics
**3d**	*Vibrio anguillarum*	①	16	Raised, round colonies with regular margins, pale yellow in color, moist and smooth, and a diameter of 1–2 mm.
	*Aeromonas bivalvium*	②	2	Slightly convex, round colonies with regular margins, moist and smooth, and a diameter of 1–2 mm.
	*Shewanella* sp.	③	2	Raised, round colonies with regular margins, translucent, orange-yellow in color, and a diameter of 1.5–2 mm.
	*Aeromonas veronii*	④	1	Round colonies with regular margins, smooth surface, pale yellow in color, and a diameter of 1–2 mm.
	*Psychrobacter* sp.	⑤	1	Round colonies with regular margins, moist and smooth, off-white in color, and a diameter of 1–2 mm.
	*Pseudomonas putida*	⑥	1	Round colonies with regular margins, moist and smooth, grayish-white in color, and a diameter of 0.5–1 mm.
	*Citrobacter freundii*	⑦	1	Raised, round colonies with regular margins, grayish-white in color, moist and smooth, and a diameter of 1–2 mm.
**5d**	*Vibrio anguillarum*	①	8	Raised, round colonies with regular margins, pale yellow in color, moist and smooth, and a diameter of 1–2 mm.
	*Aeromonas bivalvium*	②	3	Raised, round colonies with regular margins, moist and smooth, and a diameter of 1–2 mm.
	*Shewanella* sp.	③	5	Raised, round colonies with regular margins, translucent, orange-yellow in color, and a diameter of 1.5–2 mm.
	*Aeromonas encheleia*	⑧	1	Slightly convex, round colonies with regular margins, moist and smooth, grayish-white in color, and a diameter of 2–3 mm.
**15d**	*Vibrio anguillarum*	①	13	Raised, round colonies with regular margins, pale yellow in color, moist and smooth, and a diameter of 1–2 mm.
	*Aeromonas allosaccharophila*	⑨	3	Slightly convex, round colonies with regular margins, moist and smooth, grayish-white in color, and a diameter of 2–3 mm
	*Aeromonas encheleia*	⑧	1	Slightly convex, round colonies with regular margins, moist and smooth, grayish-white in color, and a diameter of 2–3 mm.

### 3.8 Histological microstructures

**Fig. 5:**
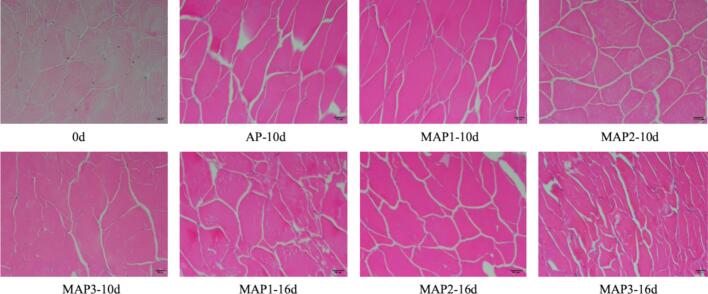
Light microscopy observations (×200) of *Trachinotus ovatus* during refrigeration.

The microstructures can reflect the quality changes of fillets. The morphology of myofibers in cross-sections of *T. ovatus* under a 200× optical microscope during refrigeration were shown in Fig. 5. On day 0, the muscle fiber of *T. ovatus* was in good condition with intact cellular structures. The fresh fillets muscle fiber bundles were compact, with clear boundaries between myofibers, tightly and orderly arranged, and small inter-fiber gaps. As storage time increased, the inter-fiber gaps in both AP and MAP of *T. ovatus* continuously enlarged, and the muscle surface structure became loose and fragmented. AP fillets were stored for 10 days, while MAP fillets were stored for 16 days. By day 10, visible cavities were observed among the myofibers in AP, accompanied by increased inter-fiber gaps, uneven arrangement, and sarcomere separation. At the same storage time, the MAP group exhibited smaller inter-fiber gaps, reduced rupture, and a more intact and dense tissue architecture compared with the AP group. As storage progressed, microbial growth and metabolism persisted, exacerbating oxidative stress and resulting in continuous protein degradation and tissue loosening (Chen et al., 2024; Qian et al., 2024). The superior performance of MAP is likely attributable to the suppression of microbial activity under specific CO₂/N₂ conditions, as evidenced by the significantly higher TVC observed in AP throughout the storage period.

### 3.9 Isolation and identification of SSO

Fish fillets stored at 0 °C were sampled on day 3, 5, and 15. Spoilage bacteria were isolated using four selective media: Iron agar medium, Nutrient agar medium, Pseudomonas selective medium, and Vibrio-specific medium. Bacterial suspensions from fish samples were sequentially diluted and plated via the spread-plating technique, followed by purification through plate streaking at least twice. Representative single colonies were successfully isolated on day 3, 5, and 15, yielding eight, five, and four distinct isolates, respectively. The morphological characteristics of these colonies are comprehensively detailed in Table 3. 10 bacterial isolates exhibited circular colony and smooth surface morphology across storage periods. Notable variations were observed in elevation, surface texture, margin regularity, and opacity. Raised colonies were dominated by *V. anguillarum* (①), *Shewanella* sp. (③) and *Aeromonas veronii* (④) at 3d (1.5–2 mm diameter), transitioning to *V. anguillarum* (①) as primary raised taxa by 15d.

Regular margins were observed in 21% of isolates (e.g., *Aeromonas bivalvium* [②]), *Shewanella* sp.(③) maintained translucency (orange-yellow pigmentation) across all time points, potentially linked to carotenoid biosynthesis under oxidative stress. Opaque colonies were predominated by *V. anguillarum* (①) and *Citrobacter freundii* (⑦) from 5d onward, correlating with biofilm formation or extracellular polymeric substance (EPS) accumulation. The temporal evolution of bacterial communities exhibited distinct successional patterns under prolonged storage conditions (3d–15d). *V. anguillarum* was the predominant taxon associated with spoilage, which demonstrated a biphasic population trend, peaking at 12 colonies (2–3 mm diameter) on day 3, declining sharply to 2 colonies on day 5, and recovering to 6 colonies (pale yellow, 1–2 mm) by day 15. This fluctuation reflects adaptive strategies to nutrient depletion and metabolic cross-talk with competitors, wherein *V. anguillarum* enhanced its stress tolerance through EPS production and biofilm formation (Costa et al., 2018).

### 3.10 16S rRNA sequencing and bacterial composition analysis

High-throughput sequencing was used to further characterize the bacterial composition and succession with Fresh, AP and MAP fillets at the end of the storage. The sequencing of fillets resulted in 111,432 paired-end sequencing reads. After double ended read splicing and screening, 98,976 effective tags with an average length of 462 bp were obtained. All those with sufficient similarity were assigned to OTUs. The analysis of OTUs, Chao1 and Ace richness estimators, Shannon index and Coverage are shown in Table 4. The overall Coverage was equal to 1.0, suggesting that all microbial phylotypes in fish fillets were identified. In addition, higher value of Chao1, Ace and Shannon index in MAP1 and MAP3 than in other treatments implied that the microbial communities of these samples had higher bacterial diversity and community evenness.

**Table 4:** Comparison of alpha diversity estimation of the 16S rRNA gene libraries by sequencing on an IlluminaHiSeq2500 platform in *T. ovatus* fillets with modified atmosphere packaging during refrigerated storage.

Samples	Clean tags	Average length (bp)	OTU	Ace	Chao1	Shannon	Coverage
Fresh	70,883	463	186	70.89	71.67	2.04	1
MAP1	109,067	462	145	115.40	113.49	2.89	1
MAP2	59,096	463	185	71.52	70.45	1.60	1
MAP3	156,416	462	142	111.60	111.38	3.05	1
AP	99,419	463	188	78.45	70.87	1.49	1

**Fig. 6:**
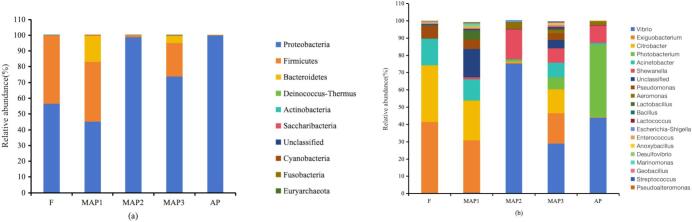
Relative abundance at the phylum (A) and genus (B) levels based on 16 s rRNA genes sequences of microbiota from *Trachinotus ovatus* fillets during refrigerated storage. F: Fresh; MAP1: 50% CO₂/50% N₂; MAP2: 70% CO₂/30% N₂; MAP3: 90% CO₂/10% N₂; AP: air packaging. *n* = 3.

Histograms were used to represent the relative abundances of the top 10 microbiota in all samples at the phylum and genus levels. As shown in Fig. 6a, Proteobacteria and Firmicutes were the major bacteria phyla in fresh sample, accounting for 56.6% and 43.4%, respectively. Zhang et al. (2019) also found that *Proteobacteria* and *Firmicutes* were predominant in vacuum or modified atmosphere packaging common carp (Zhang et al., 2019). At the end of the storage, Proteobacteria was still the dominant phylum in MAP (45.14%–98.81%) and AP (99.67%), indicating its critical role in spoilage progression under specific gas conditions. Firmicutes exhibited high abundance in MAP1 (38.00%) and MAP3 (21.44%) but drastically declined in MAP2 (1.12%) and AP (0.31%). Bacteroidetes showed moderate presence in MAP1 (16.58%) and MAP3 (4.51%), while other phyla remained below 1% across all groups.

The results for major bacterial species at the genus level are shown in Fig. 6b. *Exiguobacterium*, *Citrobacter* and *Acinetobacter* were the predominant species in fresh samples with composition proportions >15%, followed by *Pseudomonas*, *Bacillus*, *Enterococcus* and *Escherichia*-*Shigella*. *Vibrio* (43.91%), *Photobacterium* (41.97%) and *Shewanella* (10.02%) became the major microbiota in AP after 10 days, whereas *Exiguobacterium* and *Citrobacter* were reduced to negligible values, implicating the role of *Vibrio*, *Photobacterium* and *Shewanella* in quality deterioration (Chi et al., 2025; Zhu et al., 2016). Zhu et al. (2016) reported that in freshly caught large yellow croaker (*Larimichthys crocea*), *Shewanella putrefaciens* and *Vibrio* spp. constituted 29% and 22.6% of the total bacterial community, after 5 days of storage at 4 °C, the relative abundance of *Shewanella putrefaciens* increased significantly to 72.1%, whereas *Vibrio* spp. decreased to 7% (Zhu et al., 2016). For MAP, *Exiguobacterium*, *Vibrio* and *Citrobacter* were found to be the prevalent population on day 16, with an average abundance of 34.9%, 16.2% and 12.5%. This could be attributed to their facultative anaerobic metabolism, enabling adaptation to specific modified atmosphere conditions. Generally, although the bacterial composition in MAP1, MAP2 and MAP3 was different, bacterial diversity in these samples was still apparent. With regard to Fresh, *Vibrio* was found to be the prevalent population in MAP2 (75.17%) and MAP3 (28.90%), *Exiguobacterium* decreased to 30.40% and dominated in MAP1. Specially, MAP2 was mostly composed of *Vibrio* and *Shewanellaceae*, with the corresponding relative abundances of 75.2% and 17.3%, respectively.

The shifts in microbial community structure under different MAP treatments are driven by CO₂-mediated selective pressure. High CO₂ concentrations inhibit aerobic spoilage bacteria such as *Pseudomonas*, while favoring facultative anaerobic and acid-tolerant genera including *Vibrio* and *Shewanella*. In MAP2 (70% CO₂), *Vibrio* became dominant owing to its strong acid tolerance and biofilm formation ability (Flemming, 1993; Xue et al., 2022). In MAP1 (50% CO₂) and MAP3 (90% CO₂), *Exiguobacterium* predominated due to its high osmotolerance. The distinct microbial composition among treatments reflects adaptive strategies in response to CO₂-induced acidification and oxygen limitation. A non-linear relationship was observed between CO₂ level and microbial selection. *Exiguobacterium* outcompeted *Vibrio* in MAP1 via efficient fermentation, while 70% CO₂ exceeded its buffering capacity and favored acid-tolerant *Vibrio* in MAP2. In MAP3, extreme hyperosmotic stress impaired *Vibrio* viability, allowing osmotolerant *Exiguobacterium* to regain dominance (Remonsellez et al., 2018). Thus, MAP2 represents a critical threshold effect that balances general microbial inhibition and the selective survival of specific spoilage organisms. These community shifts further explain the observed differences in quality deterioration rates among treatments.

**Fig. 7:**
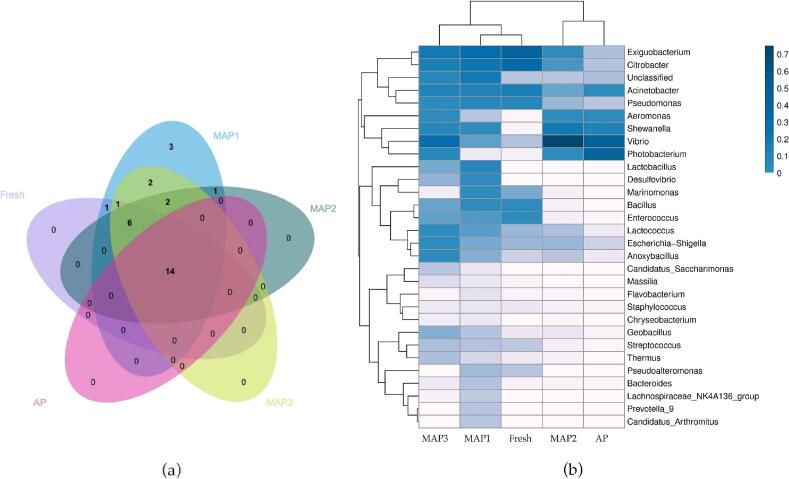
Venn diagram (a) and Community heatmap (b) analysis of microbial communities in *Trachinotus ovatus* fillets during refrigerated storage. F: Fresh; MAP1: 50% CO₂/50% N₂; MAP2: 70% CO₂/30% N₂; MAP3: 90% CO₂/10% N₂; AP: air packaging. n = 3.

The Venn diagram and the heat-map were used to show the microbiota differences among groups. As illustrated in Fig. 7a, the Venn diagram showed the unique genus distribution with shared OTUs of 30 in all samples, among which 14 common OTUs are found among the groups. Additionally, MAP1 exhibits 3 unique OTUs. With regard to community heatmap (Fig. 7b), the color represented the relative abundances of the microbial genera and the cluster trees were added on the left and top according to the similarity of genera abundances. MAP1 sample had the most diverse bacterial composition, followed by MAP3 treatment, the genera richness of MAP1 and MAP3 were obviously richer than that of MAP2 and AP whose microbiota was mainly concentrated in seven genera: *Exiguobacterium*, *Citrobacter*, *Acinetobacter*, *Aeromonas*, *Shewanella*, *Vibrio*, and *Photobacterium*.

## 4 Conclusion

MAP demonstrated comparative advantages in preservation efficacy compared to AP, maintaining significantly higher sensory scores. MAP2 and MAP3 significantly extended the shelf life of golden pompano fillets to 16 days, and MAP2 showed the best preservation performance. By contrast, MAP1 exceeded the microbiological safety limit by day 16. Notably, MAP2 exhibited the most favorable effects, prolonging the shelf life to 16 days—6 days longer than the air packaging (AP, 10 days)—thus better maintaining consumer acceptability during cold storage at 0 °C. MAP treatments effectively inhibited bacterial proliferation and delayed the deterioration of key quality markers, including TVB-N, TBARS, K-value, TVC, hardness and springiness. These results collectively confirm that MAP retards the spoilage process by slowing down protein degradation, lipid oxidation, and microbial growth. Proteobacteria persistently dominated the bacterial community across all treatment groups throughout the storage period, while MAP specifically enriched *Exiguobacterium*, *Vibrio*, and *Citrobacter* as the predominant taxa at the end of storage. Isolation and identification further revealed *V. anguillarum* as the primary specific spoilage bacteria in *T. ovatus*, highlighting their critical role in driving quality deterioration under these conditions. In summary, this study supports the use of MAP as an effective preservation technology for maintaining *T. ovatus* fillet quality under the specific 0 °C storage conditions tested. MAP2 was identified as the most suitable gas composition among the tested regimens for extending shelf life. These findings provide practical insights for the seafood industry; however, industrial applications may require further optimization based on varying supply chain parameters. Future research should focus on elucidating the mechanistic basis of MAP's antimicrobial effects and integrating multi-omics approaches to further dissect the interplay between microbial dynamics and quality attributes.”

## CRediT authorship contribution statement

**Yisheng Huang:** Writing – original draft, Methodology, Investigation, Data curation. **Zewei Zhang:** Writing – review & editing, Methodology, Investigation. **Bin Zhang:** Writing – review & editing, Methodology, Investigation. **Chang Liu:** Writing – review & editing, Investigation. **Yuzhong Zheng:** Writing – review & editing, Data curation. **Hui Zhu:** Supervision, Project administration, Methodology. **Laihao Li:** Supervision, Project administration, Methodology. **Wanling Lin:** Writing – review & editing, Supervision, Project administration, Methodology.

## Declaration of competing interest

The authors declare that they have no known competing financial interests or personal relationships that could have appeared to influence the work reported in this paper.

## Data Availability

Data will be made available on request.
